# Continuous glucose monitoring after discontinuation of incretin-based obesity pharmacotherapy: indirect evidence and a research framework for metabolic stability and weight maintenance

**DOI:** 10.3389/fendo.2026.1892632

**Published:** 2026-09-28

**Authors:** Andrijana Koceva, Andrej Janez, Mojca Jensterle

**Affiliations:** 1 Department of Endocrinology and Diabetology, University Medical Center Maribor, Maribor, Slovenia; 2 Faculty of Medicine, University of Maribor, Maribor, Slovenia; 3 Department of Endocrinology, Diabetes and Metabolic Diseases, University Medical Center Ljubljana, Ljubljana, Slovenia; 4 Faculty of Medicine, University of Ljubljana, Ljubljana, Slovenia

**Keywords:** continuous glucose monitoring, incretin-based therapy, long-term weight maintenance, obesity, semaglutide, tirzepatide, treatment discontinuation, weight regain

## Abstract

Discontinuation of incretin-based pharmacotherapy is frequent and is associated with weight regain and metabolic relapse, including deterioration in cardiometabolic risk factors. New strategies are needed to preserve metabolic stability and body weight after treatment withdrawal. Whether continuous glucose monitoring (CGM) can facilitate early detection of metabolic relapse or support long-term weight maintenance after discontinuation remains unknown. This narrative review examines the indirect evidence supporting CGM use after discontinuation of incretin-based obesity pharmacotherapy, focusing primarily on adults with obesity without established diabetes, including those with normoglycemia or prediabetes. CGM can characterize postprandial glucose responses and glycemic variability under free-living conditions while also offering real-time behavioral feedback. However, no prospective studies have established whether CGM-derived metrics precede or predict weight regain, nor whether CGM-guided interventions improve long-term weight-maintenance outcomes in this setting. Moreover, validated CGM thresholds for identifying metabolic relapse in individuals without diabetes have yet to be defined. We outline a hypothesis-generating three-phase monitoring framework for the prospective evaluation of CGM alongside body weight, appetite, dietary intake, and physical activity during the post-discontinuation transition period. If validated, unfavorable CGM trends could support earlier individualized behavioral or pharmacological intervention, before clinically meaningful weight regain or metabolic relapse occurs. Potential benefits must be balanced against costs, skin reactions, anxiety, and unnecessary dietary restrictions arising from overinterpreting unvalidated signals. CGM-guided post-pharmacotherapy care, therefore, remains investigational and requires prospective validation.

## 1 Introduction

Obesity is a chronic, heterogeneous disease characterized not only by excess adiposity but also by profound metabolic variability. Individuals with similar body mass index (BMI) may display markedly different patterns of glycemic regulation, insulin dynamics, lipid metabolism, body-fat distribution and cardiometabolic risk (1). Incretin-based obesity pharmacotherapy, including glucagon-like peptide-1 receptor agonists (GLP-1 RAs) and dual glucose-dependent insulinotropic polypeptide (GIP)/GLP-1 receptor agonists, has substantially advanced obesity pharmacotherapy by producing clinically meaningful weight loss and broad metabolic benefit.

Despite these achievements, discontinuation of incretin-based obesity pharmacotherapies is common in clinical practice, with real-world studies reporting discontinuation rates of 35–65% of patients within the first year of treatment (2, 3). Randomized withdrawal trials and follow-up studies have consistently shown that treatment withdrawal is followed by progressive weight regain and deterioration of cardiometabolic parameters (4–6). These findings reinforce the chronic nature of obesity and the importance of continued treatment or structured follow-up. However, financial constraints, limited healthcare systems reimbursement, adverse effects, treatment fatigue, medication availability, evolving clinical circumstances and pregnancy planning may necessitate treatment interruption or discontinuation. Consequently, an important clinical and research question is how to best monitor individuals after treatment withdrawal and identify patients at greatest risk of clinically meaningful weight regain and metabolic instability.

Although body-weight trajectory remains a central clinical outcome in obesity management, body weight is a cumulative measure that may not immediately capture early changes in appetite, energy intake, or glucose regulation (7). As the pharmacological effects of incretin-based therapies wane after discontinuation, changes in appetite and glycemic regulation may emerge before or alongside sustained weight regain. Available withdrawal studies have demonstrated progressive weight regain and partial reversal of cardiometabolic benefits but have not established the exact temporal sequence of all biological drivers of the relapse (7, 8). This provides a plausible rationale for investigating whether dynamic glucose patterns could complement conventional anthropometric and behavioral monitoring during the post-discontinuation period. However, it remains unknown whether changes in postprandial glucose responses or glycemic variability precede clinically meaningful weight regain or provide predictive information beyond serial monitoring of body weight, appetite, dietary intake, and physical activity.

Conventional clinical markers, including glycated hemoglobin (HbA1c), fasting plasma glucose (FPG), and oral glucose tolerance testing (OGTT), remain the established methods for diagnosing prediabetes and diabetes. However, these measurements provide valuable but static, time-point assessments of metabolic status that fail to capture day-to-day variability, postprandial glycemic fluctuations, nocturnal dysglycemia, and glucose patterns (9). Continuous glucose monitoring (CGM) enables real-time, longitudinal characterization of glycemic profiles and offers insights into postprandial responses, glycemic variability (GV), time in range (TIR), time in tight range (TITR), time above range (TAR), mean amplitude of glycemic excursions (MAGE) and temporal patterns that conventional methods cannot capture (8, 9).

Real-time visualization of glucose responses has also been explored as a behavioral support tool that may enhance awareness of the metabolic consequences of specific meals, meal timing, sedentary behavior and physical activity (8, 10). Its relevance may be especially pronounced during high-risk transitional phases in obesity management, including after discontinuation of incretin-based therapies and during the periconceptional window.

In this narrative review, we critically evaluate indirect evidence regarding CGM use as a potential strategy for long-term metabolic stability and weight maintenance in personalized care after discontinuation of incretin-based obesity pharmacotherapy, focusing primarily on adults with obesity without established diabetes, including those with normoglycemia or prediabetes. We frame the conceptual rationale by summarizing the metabolic basis of post-discontinuation weight regain, characterizing CGM as a phenotyping and behavioral feedback tool, focusing on the post-discontinuation transition as the highest-yield clinical context, and additionally consider the parallel scenario of pre- and periconception care, where women must discontinue incretin-based therapy ahead of planned pregnancy. We close with a hypothesis-generating framework and a proposed proof-of-concept study design through which the predictive and interventional value of CGM could be prospectively evaluated.

This narrative review was informed by a PubMed search from database inception to July 2026. Search terms included combinations of continuous glucose monitoring and its derived metrics (time in range, TIR, time in tight range, TITR, time above range, TAR, glycemic variability, GV, mean amplitude of glycemic excursions, MAGE, postprandial glucose) with terms related to obesity and glycemic status (obesity, overweight, prediabetes, normoglycemia, intermediate hyperglycemia). These terms were combined with terms related to incretin-based obesity pharmacotherapy (GLP-1 receptor agonist, GLP-1/GIP receptor agonist, semaglutide, tirzepatide, liraglutide) and its discontinuation (discontinuation, withdrawal, weight loss, weight regain, weight maintenance, relapse) and terms related to behavioral and dietary applications (behavioral tool, physical activity, diet composition, carbohydrate intake, appetite, eating patterns) and preconception related terms (preconception, periconception, pregnancy, maternal obesity, gestational weight gain, gestational diabetes). English-language publications involving adults were screened by title and abstract for relevance. Reference lists of eligible publications were searched manually to identify additional sources.

## 2 Transition following the incretin-based therapy discontinuation: a challenge for long-term obesity care

The clinical effectiveness of incretin-based therapies in inducing and maintaining weight loss is well established, but their benefits are largely conditional on continued exposure. In the STEP 4 trial, participants who continued semaglutide 2.4 mg weekly treatment maintained and extended their initial weight loss, whereas those switched to placebo progressively regained a substantial proportion of lost weight within one year (4). A similar pattern was observed in the SURMOUNT-4 trial, in which continued tirzepatide treatment maintained weight reduction, while treatment withdrawal was followed by weight regain (5). However, individual trajectories vary, and it is not established which markers can reliably identify individuals at greatest risk of clinically meaningful regain.

Mechanistically, weight regain after incretin-based therapy withdrawal may reflect multiple interacting adaptations, including a reduction in central anorexigenic signaling and the re-emergence of orexigenic drive, a partial reversal of treatment-induced improvements in insulin sensitivity and beta-cell function, attenuated thermogenic responses, and behavioral re-exposure to a permissive food environment. Together, these adaptations re-establish the energy-conservation phenotype that characterized the pre-treatment state. However, the temporal sequence of these adaptations after incretin discontinuation has not been fully characterized. A *post hoc* analysis of SURMOUNT-4 demonstrated that in participants with obesity without diabetes after tirzepatide discontinuation, HbA1c and fasting glucose began to diverge from the continuation arm within the first 16 weeks and subsequently largely plateaued, while body weight continued to increase through week 88. The steepest early rise in glycemic levels was observed among those who ultimately regained the most weight (11). Because incretin-based therapies preferentially reduce postprandial glucose excursions through mechanisms including glucose-dependent insulin secretion, glucagon suppression, and delayed gastric emptying, loss of these acute glycoregulatory effects may plausibly occur more rapidly after withdrawal than reversal of central appetite-suppressive and hormonal signaling governing caloric intake, though this differential effect has not been directly demonstrated (12). The resulting early hyperglycemia and hyperinsulinemia could reinforce positive energy balance and contribute to the self-perpetuating weight regain, but this mechanism also remains a proposed explanation rather than one established through controlled human data (13). Lastly, the role of postprandial glycemic exposure is supported by dietary intervention studies in non-pharmacological weight maintenance trials, where higher postprandial glycemia has been associated with impaired weight-loss maintenance and glycemic status (14). Nevertheless, this should be regarded as a biologically plausible mechanism rather than directly confirmatory in the setting of incretin therapy discontinuation.

From a monitoring perspective, several of these adaptations may be detectable by CGM in glucose dynamics (rising postprandial excursions, decreased TIR or TITR, increased TAR, increased GV), possibly before they become detectable in body weight (8, 15, 16). This potential dissociation between metabolic and anthropometric trajectories is exactly the gap that CGM is positioned to fill. Whereas a clinical visit at three- or six-month intervals captures only the cumulative outcome, such as weight regain or rising HbA1c, CGM samples glucose every few minutes under free-living conditions and could characterize early glycemic trajectories between clinical visits. Whether these changes precede measurable weight regain or provide incremental predictive value beyond serial weight and behavioral monitoring remains unknown and constitutes the central hypothesis that requires prospective testing. In the context of a chronic disease with limited reimbursement windows for pharmacotherapy, the ability to detect early metabolic relapse and act on it has direct therapeutic and public health value.

## 3 CGM as a tool for metabolic phenotyping in obesity without diabetes

The clinical interpretation of CGM-derived metrics depends strongly on baseline glycemic status. Accordingly, we consider adults without diabetes, adults with obesity and normoglycemia, and adults with prediabetes or intermediate hyperglycemia separately. CGM provides descriptive information on interstitial glucose patterns but is not currently validated for diagnosing prediabetes or diabetes, and abnormal patterns in individuals without diabetes should be regarded as screening or monitoring signals that require clinical interpretation and laboratory confirmation.

### 3.1 Reference patterns and physiological glycemic heterogeneity in adults without diabetes

Substantial TIR dispersion can be observed at similar HbA1c values in adults without diabetes, suggesting that individuals with comparable average glycemia may exhibit markedly different glycemic patterns, reflecting underlying metabolic heterogeneity (9, 17).

In a large CGM study of adults without diabetes aged 40 to 70 years, mean fasting interstitial glucose was 96.2 mg/dL (5.3 mmol/L) and rose by 0.23 mg/dL per year of age (0.013 mmol/L per year), with intraindividual day-to-day variability expressed as a standard deviation (SD) of 7.52 ± 4.31 mg/dL (0.42 ± 0.24 mmol/L). Notably, among participants whose first fasting glucose value was within normal range, 40% subsequently recorded values within the prediabetes range during repeated measurements, illustrating the biological variability of fasting glucose and the limitations of relying on either a single measurement or an isolated CGM value (18). Large cohort studies of over 7000 non-diabetic adults further demonstrated substantial inter- and intra-individual variability in glucose patterns, with associations with age, sex, sleep, diet and adiposity (19). In the community-based Framingham Heart Study, participants classified as normoglycemic according to laboratory fasting glucose and HbA1c spent almost 98% of time within the standard 70–180 mg/dL (3.9-10.0 mmol/L) range, but only 87% of time within the tighter 70–140 mg/dL (3.9-7.8 mmol/L) range, variability occurring entirely within the euglycemic range and invisible to HbA1c and FPG (20).

CGM has demonstrated substantial inter-individual variability in postprandial glucose responses among normoglycemic individuals with excursions differing markedly even following identical meals, likely due to multiple factors, such as carbohydrate sensitivity, meal timing and other individual characteristics such as age (21, 22). These observations are particularly relevant to the post-discontinuation monitoring. A single increase in postprandial glucose or TAR may therefore reflect meal composition, meal timing, physical activity, stress, intercurrent illness, medication change, or sensor-related variation. Consequently, candidate CGM signals of post-discontinuation metabolic relapse would need to represent persistent changes across repeated monitoring periods rather than isolated excursions.

### 3.2 CGM profiles in obesity with normoglycemia

Against this background, observational evidence suggests that individuals with obesity may exhibit clinically relevant alterations in glucose profiles despite having FPG and HbA1c within the normal range. Normoglycemic individuals with obesity spend less time in the TITR (70–140 mg/dL or 3.9-7.8 mmol/L) compared to normoglycemic peers without obesity (83.7% vs. 89.0%), again despite comparable HbA1c and FPG (20). Normoglycemic individuals with class III obesity have been shown to exhibit substantially greater GV than normal-weight peers, with MAGE nearly twice as large (48.6 vs 26.3-28.3 mg/dL or 2.7 vs. 1.5-1.6 mmol/L) and time above 140 mg/dL (7.8 mmol/L) forty-fold greater (12% vs. 0.3%), despite normal OGTT, HbA1c and FPG (23). Similarly, in a study of middle-aged men with obesity without diabetes, CGM revealed that frequent glucose elevations exceeded thresholds commonly associated with dysglycemia, and found associations between specific lifestyle behaviors and elevated peak interstitial glucose levels, with more frequent snacking and lower daily step counts being significantly associated with a higher likelihood that peak sensor glucose values would reach or exceed 200 mg/dL (11.1 mmol/L) (24).

Additional mechanistic evidence comes from a pilot cross-sectional study of 37 adults with obesity, including 20 with normoglycemia and 17 with type 2 diabetes (25). In the normoglycemic subgroup, mean CGM glucose was inversely associated with OGTT-derived insulin sensitivity (r= -0.51, p=0.02) and clamp-derived glucose infusion rate (r= -0.75, p=0.001), whereas CGM-derived metrics were not associated with HbA1c. These findings suggest that mean CGM glucose may reflect insulin-resistance-related metabolic heterogeneity not captured by HbA1c in adults with obesity and normoglycemia. However, the study was small, cross-sectional, and did not evaluate longitudinal changes, progression to dysglycemia, or weight gain (25).

The largest characterization to date, an observational study of 8687 adults with normoglycemia, found that participants spent a median of 93% of TITR (70–140 mg/dL or 3.9-7.8 mmol/L) and 95% within TIR (70–180 mg/dL or 3.9-10.0 mmol/L), and that time-in-range metrics correlated with 45 metabolic phenotypes. More time above > 140 mg/dL was associated with higher waist circumference, blood pressure, triglycerides, lower HDL, visceral fat, and liver attenuation. Notably, greater time above TIR or TITR was prospectively associated with incident metabolic disease over a mean of 2.6 years, with a hazard ratio of 1.21 and 1.34, respectively (26). Nevertheless, this preliminary observational evidence does not establish causality, diagnostic validity, or a validated threshold for metabolic relapse after weight loss.

Taken together, CGM may characterize glucose patterns associated with adiposity and lifestyle exposures that are not reflected by conventional measurements alone. However, the available studies are predominantly small, cross-sectional, and heterogeneous in participant selection, monitoring duration, and dietary conditions.

### 3.3 CGM profiles in obesity with prediabetes

Differences in CGM-derived metrics, including TIR, time above range (TAR) and indices of glycemic variability (GV) may capture early metabolic stress along the normoglycemia-prediabetes-type 2 diabetes continuum (17, 27). Prediabetes is increasingly conceptualized as a heterogeneous stage within a continuum of progressive dysglycemia rather than as a uniform category. A recently proposed three-stage framework published in Lancet Diabetes & Endocrinology describes stage 1 as increased metabolic risk with glycemia remaining within the conventionally normal range, stage 2 as intermediate dysglycemia, with distinction between slow and rapid progression, and stage 3 as overt type 2 diabetes. CGM-derived phenotypes, including TITR, have been proposed as potential tools for refining this staging. However, this framework remains conceptual, and no CGM thresholds have been validated for assigning individuals to these stages (28).

CGM-derived measures of glycemic variability are consistently elevated in prediabetes compared with normoglycemia, correlating with defects in first-phase insulin secretion and reflecting beta-cell dysfunction that precedes overt diagnostic thresholds (21, 27, 29). A recent systematic review and meta-analysis of 16 studies found that prediabetes was associated with a higher MAGE, greater TAR, and a higher 24-hour mean glucose than normoglycemia (30), supporting the potential of CGM to complement conventional metabolic risk assessment in this population. Interventional studies further support CGM utility in this population as randomized trials combining CGM with individualized nutrition therapy in individuals with obesity and prediabetes demonstrated improvements in TIR, TAR, mean glucose, and dietary quality, including increased whole-grain and plant-based protein intake (31, 32). This is directly relevant to the incretin-based obesity pharmacotherapy discontinuation period as therapy cessation may be accompanied by a progression along the dysglycemia continuum, and CGM may detect the metabolic shift of this trajectory earlier than HbA1c or FPG alone, though this remains to be prospectively established.

Taken together, these findings suggest that CGM may serve as a tool for metabolic phenotyping in obesity, enabling the identification of subclinical dysglycemia, characterization of inter-individual variability, and improved understanding of early metabolic disturbances not captured by traditional methods. This phenotyping role is directly relevant to the post-discontinuation setting, in which baseline metabolic heterogeneity could influence subsequent glycemic and weight trajectories. Nevertheless, across all these groups, it must be emphasized that no CGM-derived thresholds have been validated to define metabolic relapse in obesity without diabetes. Current targets are extrapolated from diabetes and require prospective, outcome-guided validation before they can guide clinical decisions in this setting.

## 4 CGM as a tool for behavioral and dietary insight

Beyond metabolic characterization, CGM has also been explored as a tool to support behavioral and dietary modification. Research in behavioral science has shown that biological feedback engages motivation and reward systems, particularly during the early phase of behavior change, when motivation is high. The proposed additional value of CGM over conventional monitoring of glucose metabolism is its capacity to provide immediate, objective, and personalized biological feedback that links daily behaviors to glucose responses. Biological feedback may help individuals recognize the consequences of daily behaviors, strengthen motivation and self-regulation and facilitate rapid adjustment of eating and physical activity patterns (33, 34). By providing real-time information and visualizations of glucose responses, CGM may enable individuals to directly observe the metabolic consequences of specific foods, meal timing, and physical activity, increasing awareness and enabling them to make more informed decisions in daily life (8, 35). Although this mechanism may be relevant following discontinuation of incretin-based therapies, no studies have evaluated whether CGM-based behavioral feedback improves weight maintenance in this setting.

Several studies suggest that CGM-based feedback may influence dietary composition. In individuals with impaired glucose tolerance, higher GV was associated with daily consumption of carbohydrate and refined grains, while lower GV was associated with whole grains and daily protein intake (36). Integration of CGM into lifestyle programs has been associated with reduced intake of high-glycemic index foods, lower carbohydrate consumption, and increased dietary fiber over time (35). This suggests that real-time glucose data may support more informed dietary choices by linking behavior to measurable metabolic responses.

Supporting this behavioral rationale, a large observational study of adults without diabetes or prediabetes (n=3634) found that higher CGM-derived time in range was associated with lower HbA1c, lower carbohydrate intake and higher protein intake, and that shorter sleep duration correlated with higher mean glucose, indicating that CGM metrics reflect diet and lifestyle even in normoglycemic individuals (37). However, these exploratory associations do not demonstrate that CGM feedback caused behavioral change. In 168 individuals with intermediate hyperglycemia, a 12-week multicomponent lifestyle intervention combining a smartphone application, lifestyle support and CGM increased time in the tight range (TITR) and was accompanied by reduced carbohydrate intake and weight loss (38). Nevertheless, because CGM was only one component of the intervention, its independent contribution cannot be determined.

Eating behavior extends beyond dietary composition and also includes patterns such as meal frequency, timing, and snacking, collectively referred to as eating patterns. In a cross-sectional study of adults at increased risk of type 2 diabetes, a greater number of daily eating occasions, particularly increased snacking frequency, was associated with greater adiposity and less favorable glycemic profiles assessed by CGM (39). These findings suggest that the daily distribution of food consumption may also play a distinct role in metabolic regulation, and that CGM may capture the glycemic impact of such eating patterns in real time, enabling targeted behavioral modifications.

Most interventional evidence relating CGM to physical activity is derived from individuals with type 2 diabetes and should therefore be considered indirect evidence for adults with obesity without diabetes. In a pilot study of sedentary adults with overweight or obesity, CGM use over a 10-day self-monitoring period was highly acceptable and associated with improved readiness for physical activity (10). In a randomized study, participants receiving CGM-informed physical activity counseling alongside standard diabetes education demonstrated greater physical activity self-efficacy and confidence in maintaining physical activity and resisting relapse, increased moderate-intensity activity minutes, reduced sedentary time, and significantly lower HbA1c and BMI compared with those receiving standard diabetes education alone (40). Similarly, a randomized trial in individuals with poorly controlled type 2 diabetes found that intermittent CGM use was associated with significantly greater increases in total weekly exercise time compared with self-monitoring of glucose (+158.4 vs. +43.5 minutes per week; p = 0.02), alongside reductions in caloric intake, body weight and glycemic variability as measured by MAGE (41).

More broadly, a systematic review and meta-analysis of 25 randomized trials involving 2996 participants found that interventions incorporating CGM-based feedback modestly reduced HbA1c and increased TIR but did not significantly improve TAR, body weight and BMI. Dietary and physical activity outcomes were reported in only four and five trials, respectively and could not be pooled because of heterogeneous outcome measures. Most trials involved people with type 2 diabetes, with only three trials conducted in populations categorized as having obesity, and all interventions were multicomponent, limiting direct applicability to adults with obesity without diabetes and preventing attribution of the observed effects to CGM alone (42). Notably, some randomized trials have also demonstrated a legacy effect of CGM use, with improvements in glycemic control persisting months after device discontinuation, suggesting that CGM may support durable behavioral change beyond the period of active monitoring (15). This durability is of particular interest in the context of post-discontinuation weight maintenance, where the goal is precisely to sustain behavior beyond an active intervention window.

Several caveats are important. Most studies on behavioral feedback have involved participants with type 2 diabetes, in whom baseline dysglycemia, diabetes duration, and glucose-lowering medications influence CGM metrics independently of body weight, thereby providing greater scope for improvement in glycemic parameters. CGM-related effects are typically observed within structured multicomponent interventions, and the device’s independent contribution, in isolation from coaching and education, is difficult to discern. The potential additional value of CGM monitoring compared with regular weight monitoring, appetite, dietary intake and physical activity assessment lies in providing immediate feedback linking behaviors with glucose responses. However, whether this translates into better long-term outcomes remains unknown.

Potential disadvantages include cost, skin reactions, CGM-related anxiety or misinterpretation of normal glucose fluctuations (43). CGM measurements should therefore always be interpreted alongside assessments of body weight, appetite, dietary intake, and physical activity. CGM should not be considered a stand-alone intervention but rather a tool that may facilitate behavioral change when combined with structured education, coaching, and individualized feedback. This positioning is decisive when designing post-discontinuation transition pathways.

## 5 CGM after incretin-based therapy discontinuation: a hypothesis-generating research framework

### 5.1 Rationale and current evidence

While body weight reduction is typically used as a primary outcome in obesity management, changes in glucose regulation and GV may occur earlier and provide additional insight into treatment response. Lifestyle interventions, including dietary modifications and increased physical activity, may result in rapid improvements in postprandial glucose responses and GV, even in the absence of significant weight loss (44, 45). Although data specifically examining CGM use following discontinuation of anti-obesity pharmacotherapy remains limited, emerging perspectives suggest that CGM may help contextualize changes in eating patterns, meal timing and glycemic responses during pharmacological treatment (8) as well as provide insights into inter-individual variability in treatment response (16).

Evidence from metabolic surgery provides a related but distinct example. In individuals with obesity undergoing metabolic surgery, CGM has revealed substantial inter-individual variability in glycemic patterns despite similar HbA1c values (46). Additionally, in individuals following metabolic surgery, CGM has been shown to be particularly useful for detecting postbariatric hypoglycemia, identifying significant hypoglycemic events in over 50% of individuals who appeared normoglycemic on standard mixed-meal testing (15). However, these studies demonstrate the ability of CGM to detect glycemic abnormalities after metabolic surgery but do not establish that CGM-derived changes predict subsequent weight regain or that a CGM-guided intervention could improve weight-maintenance outcomes.

In a general post-weight-loss setting, the OWL-CGM trial was a 24-week, randomized, assessor-blinded feasibility trial involving 49 adults without diabetes who had intentionally lost more than 5 kg during the preceding 6 months and had a BMI>25 kg/m^2^ before weight loss. Participants were randomized to a weight-maintenance program alone or a weight-maintenance program combined with CGM-based feedback. Both groups received individualized dietary and physical activity guidelines and consultations with a dietitian, while the CGM group additionally received guidance on applying glucose feedback to dietary and physical activity behaviors. Among completers, body weight decreased by 2.7 ± 1.1 kg in the CGM group but increased by 0.5 ± 0.8 kg in the control group (p=0.032). The CGM group also achieved a greater reduction in energy intake. Although these findings support the feasibility and potential interventional value of adding CGM to structured weight-maintenance care, the study was small, CGM was part of a multicomponent intervention, and the trial did not assess the predictive value of CGM or weight regain following discontinuation of incretin-based obesity pharmacotherapy (47).

Periods following the discontinuation of incretin-based therapies represent a critical yet understudied phase in obesity management. In the STEP-1 trial extension, participants who discontinued semaglutide after 68 weeks regained approximately two-thirds of their prior weight loss within one year, alongside reversal of improvements in cardiovascular risk (48). Similarly, the STEP-4 and SURMOUNT-4 trials demonstrated that discontinuation of semaglutide or tirzepatide resulted in significant progressive weight regain compared to continued treatment (4, 5). A recent systematic review and meta-analysis of 18 randomized controlled trials quantified this metabolic rebound comprehensively, demonstrating that incretin-based obesity pharmacotherapy discontinuation in individuals with obesity was associated with a mean body weight gain of 5.63 kg and a mean increase in HbA1c of 0.25%, accompanied by concurrent deterioration in blood pressure and lipid parameters (12). Furthermore, a systematic review characterizing the trajectory of post-cessation weight regain demonstrated a predictable, decelerating pattern, with approximately 60% of lost weight regained within one year, suggesting that the most rapid and clinically significant rebound occurs in the early weeks to months following discontinuation, precisely the window during which real-time metabolic monitoring would be most actionable (49).

Consistent with this decelerating pattern, real-world follow-up data indicate that the magnitude of weight increase in the first weeks after treatment cessation is itself a significant predictor of subsequent regain, suggesting that the earliest post-discontinuation changes carry prognostic information and reaffirming the rationale for concentrating monitoring in this early window (50). A *post hoc* analysis of SURMOUNT-4 showed that deterioration of HbA1c and fasting serum glucose after tirzepatide withdrawal tracked the magnitude of weight regain, showing that patients with minimal regain retained most of their improvement, while participants who regained ≥ 75% of weight, cardiometabolic parameters returned to baseline by week 88 (11).

Although direct evidence on CGM use following treatment discontinuation is limited, CGM’s ability to capture dynamic, real-time changes in glucose regulation strongly supports its potential utility during this transition. Following discontinuation of incretin-based obesity pharmacotherapy, the pharmacological suppression of appetite and modulation of gastric emptying are removed, and dietary behavior, meal composition and postprandial glycemic responses may shift rapidly. Changes in postprandial glucose responses, GV, TITR or TAR may occur before clinically detectable weight regain, reflecting alterations in dietary behavior, appetite control, and insulin sensitivity. The proposition that CGM could identify metabolic deterioration before clinically meaningful weight regain after discontinuation of incretin-based therapies should be regarded as a testable hypothesis. Early identification of these adverse metabolic changes could enable timely, individualized intervention, reinforcement of dietary and physical activity strategies, structured behavioral support, dose adjustment if pharmacotherapy is ongoing, or reinitiation or escalation of pharmacotherapy when permitted by clinical and reimbursement context.

A single static measurement taken at one point during the post-discontinuation transition period can be misleading. If fasting glucose or HbA1c is measured before the drug’s clearance, the result may appear reassuring even if the underlying trajectory is already deteriorating, creating a false sense of security (51). Conversely, isolated reading cannot distinguish a transient excursion from the onset of sustained metabolic drift. Serially repeated monitoring across the full washout and early post-discontinuation window, by contrast, captures the direction and slope of change and may be better suited to detecting emerging metabolic relapse before it is reflected in body weight or in intermittently sampled markers.

### 5.2 A proposed framework for CGM-guided post-pharmacotherapy care

Direct evidence on CGM specifically during the post-discontinuation transition is currently lacking. The proposed framework below should therefore be regarded as hypothesis-generating and intended to inform future prospective evaluation. Its primary target population is adults with obesity who discontinue incretin-based therapy and do not have diabetes.

During the pre-discontinuation phase, a 14-day CGM wear could establish the individual’s reference glycemic profile during stable on-treatment status, characterizing TIR, TITR, TAR, MAGE, and the postprandial-response signature to habitual meals. This will serve as the personalized comparator for subsequent assessments.

In the early post-discontinuation phase (weeks 0–12 after withdrawal), repeated CGM wears at predefined intervals, for instance, at weeks 4, 8, and 12, capture early drift in the candidate CGM markers, such as postprandial excursions, TAR, TITR, CV or MAGE. These time points are pragmatic rather than validated. Week 4 is selected to capture early post-withdrawal changes after substantially declining drug exposure. This is supported by observational evidence that weight change during the first four weeks after tirzepatide cessation is associated with subsequent weight regain (50). Weeks 8 and 12 provide equally spaced assessments during the first three months, allowing estimation of the direction and trajectory of within-person change during the early post-withdrawal period, where most circulating drug exposure declines and early changes in weight and conventional glycemic markers become detectable. Complementary evidence from the *post hoc* analysis of SURMOUNT-4 showed that reversal of improvements in HbA1c and fasting serum glucose was detectable within the first 16 weeks after tirzepatide withdrawal and was greater among participants who subsequently regained more weight, supporting a focus on the early post-discontinuation period (11).

In the longer-term maintenance phase (months 3–12 and beyond), intermittent CGM wears every three to six months, combined with body weight and behavioral assessments, may support sustained engagement and provide objective evidence to inform reinitiation of pharmacotherapy if relapse criteria are met. Repeated CGM measurements should be analyzed alongside the percentage of weight regain and OGTT and HbA1c as confirmatory testing. Throughout, CGM data are interpreted alongside structured behavioral support, dietary counseling, and physical-activity prescription, in line with the consistent finding that CGM works as a *tool*, not as a stand-alone intervention.

Defining *relapse criteria* on CGM in the post-pharmacotherapy obesity population is a research priority. Any abnormal finding in this setting should be treated as a screening signal that warrants confirmation, not as a diagnostic result. Plausible candidate signals include sustained increases in TAR above the individual baseline, expansion of MAGE beyond pre-discontinuation values, reduction in TITR, and re-emergence of high-amplitude postprandial peaks in response to standardized meals. Quantitative thresholds will need prospective validation, but the principles align with both the broader CGM-in-obesity literature (15–17, 20, 21, 23, 24, 27, 29, 30) and with international consensus on CGM data interpretation (9).

Interpreting these signals requires distinguishing a genuine relapse trajectory from normal within-person variability. An isolated fluctuation in postprandial glucose would be insufficient as it may reflect meal composition and timing, sleep, physical activity, stress, illness, medication changes, or sensor-related variation rather than metabolic relapse (18, 22, 37). To reduce dietary confounding, interpretability improves substantially when the postprandial response is assessed relative to a standardized meal rather than to free-living intake. Lastly, in normoglycemic adults, the cumulative time spent above the upper limit of TITR (> 140 mg/dL or 7.8 mmol/L) may provide greater resolution than either an isolated postprandial peak or the conventional TIR (26). Taken together, CGM-derived metrics should therefore be interpreted as a trend across repeated assessments rather than from any single reading, and always in relation to body weight, dietary intake, appetite, and physical activity, so that a sustained multimodal shift triggers clinical action.

**Figure 1** shows the proposed hypothesis-generating framework for CGM-guided assessment after discontinuation of incretin-based therapy.

**Figure 1:**
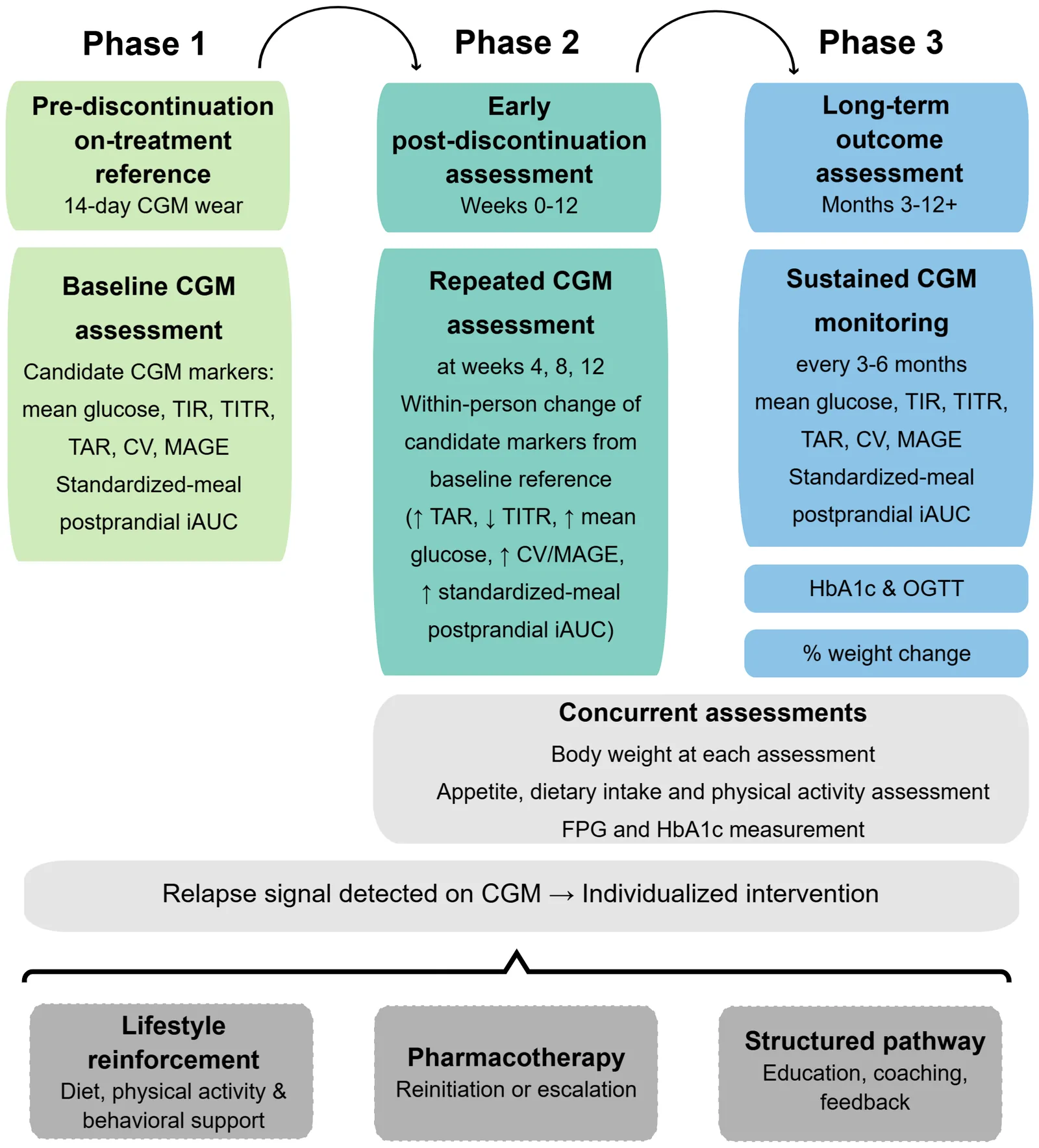
Hypothesis-generating research framework for CGM assessment after discontinuation of incretin-based obesity pharmacotherapy. The proposed monitoring intervals and intervention signals have not been clinically validated and should not be interpreted as clinical recommendations.

### 5.3 Proposed proof-of-concept study

A suitable proof-of-concept evaluation would be a multicenter, prospective cohort study in adults with obesity without established diabetes who discontinue semaglutide or tirzepatide treatment after documented weight loss of at least 5%. Participants with normoglycemia and prediabetes would both be included as prespecified subgroups, with analysis stratified by baseline glycemic status. Before treatment discontinuation, all participants would undergo a 14-day blinded CGM assessment to establish an on-treatment reference profile, along with standardized measurements of body weight, appetite, dietary intake, physical activity, sleep, fasting plasma glucose, and HbA1c. CGM assessments would be repeated at weeks 4, 8 and 12 and at 6 and 12 months, with CGM data remaining blinded to participants and treating clinicians. To reduce dietary confounding, a standardized test meal would be administered at each CGM assessment, with the postprandial incremental area under the curve analyzed alongside free-living variability metrics.

The primary outcome would be the proportion of treatment-induced weight loss regained by month 12, and the principal analysis would assess whether within-person changes in a prespecified set of CGM metrics during weeks 4-12, including mean glucose, TIR, TITR, TAR, coefficient of variation, and MAGE, improve the prediction of subsequent weight regain. Secondary predictive outcomes would include the proportion of participants with at least 5% weight gain from discontinuation weight by month 12, associations of individual CGM metrics with changes in fasting plasma glucose and HbA1c. Appetite, dietary intake, physical activity, and sleep would be analyzed as explanatory variables. Process outcomes would include recruitment, retention, completion, and data completeness, acceptability of repeated sensor wear, and sensor-related skin reactions.

If CGM demonstrated incremental predictive value beyond conventional monitoring, a subsequent randomized trial should compare the CGM-guided intervention pathway with an intensity-matched structured follow-up to determine whether CGM-guided care reduces weight regain after discontinuation of incretin-based obesity pharmacotherapy. Both groups would follow the same monitoring schedule, but CGM results would be available to participants and clinicians only in the intervention group, with the control group remaining blinded. Interventional triggers would be prespecified based on the prospective predictive study. The primary interventional outcome would be the proportion of treatment-induced weight loss regained by month 12, with secondary evaluation of cardiometabolic outcomes, adverse effects, acceptability, and cost effectiveness.

This staged approach would establish predictive value without presuming clinical effectiveness and reserve evaluation of interventional value for the subsequent randomized trial.

## 6 CGM in the preconception period: a parallel high-risk transition

A second high-risk discontinuation scenario, conceptually parallel to the post-pharmacotherapy setting, is the preconception period in women undergoing obesity treatment with incretin-based therapies.

Obesity in women of reproductive age is associated with subfertility, increased risk of miscarriage, gestational diabetes, hypertensive disorders in pregnancy and adverse neonatal outcomes (52, 53). Beyond immediate pregnancy complications, the intrauterine metabolic environment shaped by maternal obesity and hyperglycemia may have long-lasting consequences for offspring cardiometabolic health (53). The preconception and periconception periods, therefore, represent critical windows during which maternal metabolic status may influence fertility, pregnancy outcomes, and long-term offspring health through epigenetic, cellular and metabolic mechanisms (52, 54, 55). In this context, optimizing maternal metabolic status before conception is widely recommended and is a core element of preconception care (53, 54).

Incretin-based therapies are increasingly used for weight and glycemic management in women of reproductive age. However, because of animal-toxicity signals and current regulatory guidance, these agents must be discontinued before planned pregnancy. In line with product labeling, semaglutide should be discontinued at least 2 months before intended conception, and tirzepatide at least 1 month before conception (51). These intervals reflect their long elimination half-lives, approximately 7 days for semaglutide and 5 days for tirzepatide. Semaglutide, therefore, remains quantifiable in plasma for approximately 5–7 weeks after the last dose, whereas tirzepatide is largely eliminated within 4 weeks, supporting a shorter washout (56, 57). Discontinuation of these therapies outside of pregnancy is well established to result in weight regain and worsening of glycemia (4, 5). In a large retrospective electronic record-based cohort composed of primarily women with obesity, GLP-1 RA discontinuation before or during early pregnancy was associated with greater gestational weight gain (13.7 kg vs 10.5 kg) and higher rates of gestational diabetes (20% vs. 15%), hypertensive disorders in pregnancy (46% vs. 36%) and preterm delivery (17% vs. 13%) compared with propensity-matched unexposed pregnancies (58). These findings support the need for structured transition support and prospective research, but do not demonstrate that treatment withdrawal itself caused the observed adverse outcomes as residual confounding and confounding by indication remain possible (59).

The potential role of CGM in this setting is unproven but could have a monitoring role. Given the established association between maternal glycemic profiles during pregnancy and offspring outcomes, ensuring metabolic stability in the periconception period through tools capable of dynamic real-time monitoring may represent an additional layer of individualized care (55, 60). By capturing early metabolic alterations following treatment discontinuation, before clinically detectable weight regain or adverse glycemic changes manifest through a change in conventional markers, CGM may enable earlier identification of metabolic relapses and timely intervention, whether through reinforcement of lifestyle strategies, dietary counseling, or reconsideration of pharmacological approaches in coordination with obstetric and endocrine care.

## 7 Evidence gaps, research priorities and policy considerations

Several gaps must be addressed before CGM-guided post-pharmacotherapy obesity care can be operationalized. The supporting evidence is indirect and heterogeneous, encompassing adults without diabetes, individuals with prediabetes or type 2 diabetes. None of these groups directly represents the primary target population of adults with obesity without diabetes after discontinuation of incretin-based therapy. The findings should therefore not be considered directly transferable to this setting.

Moreover, clinically meaningful thresholds for TIR, TITR, TAR, glycemic variability or MAGE have not been validated for detecting metabolic relapse in this setting. Available TIR/TITR/TAR/MAGE targets derive predominantly from diabetes populations and cannot be directly extrapolated to adults with obesity without diabetes. Shah et al. recently proposed a conceptual staging for the normoglycemia-prediabetes-type 2 diabetes continuum that incorporates TITR (70–140 mg/dL or 3.9-7.8 mmol/L) as a potential adjunct measure. However, the proposed TITR ranges remain preliminary and require prospective validation.

Before candidate CGM metrics can be considered clinically meaningful, a first prospective proof-of-concept study should establish whether relatively within-person changes in these metrics are associated with subsequent weight regain and provide predictive value beyond conventional monitoring. If predictive value is demonstrated, a subsequent randomized interventional trial should determine whether a CGM-guided pathway improves outcomes compared to structured standard follow-up in the target population. Relevant outcomes should include sustained weight maintenance, recurrence of cardiometabolic risk, quality of life and healthcare utilization. Trial designs are needed that compare structured CGM-guided pathways against usual-care follow-up, with pre-specified intervention triggers, including reinforcement of behavioral support and reinitiation of pharmacotherapy.

To our knowledge, the cost-effectiveness of this approach in adults with obesity without diabetes discontinuing incretin-based therapy is currently unknown. Although CGM has been reported to be cost-effective in selected diabetes populations, these findings are highly context-dependent (17, 61). A lifetime microsimulation from a US payer perspective estimated that adding CGM to ongoing GLP-1 receptor agonist therapy was cost-effective in adults with type 2 diabetes, including individuals not using intensive insulin. However, the model assumed a CGM-associated reduction in HbA1c and cannot be extrapolated to intermittent post-discontinuation monitoring in adults with obesity without diabetes (61). Health-economic analyses must address access, reimbursement, and cost-effectiveness when CGM use extends across years rather than weeks. In the periconception window, prospective data integrating CGM with obstetric outcomes after incretin-based therapy discontinuation are needed to refine the recommendations of recent reports. From a policy perspective, time-limited reimbursement of incretin-based therapy may leave patients at a predictable risk of weight regain and clinicians with limited evidence-based strategies for its early detection or mitigation. Integration of CGM into post-pharmacotherapy obesity care would require evidence of clinical benefit over standard monitoring, validated interpretation and intervention pathways, and demonstration of cost-effectiveness. Embedding such pathways into obesity care will require alignment between clinical guidelines, payer policy, and prospective evidence generation.

## 8 Conclusion

Continuous metabolic monitoring offers a potential dynamic perspective on obesity beyond body weight alone. CGM characterizes real-time glycemic patterns and may warrant investigation during high-risk transitional periods, including after incretin-based therapy discontinuation and in the preconception window. CGM could potentially provide complementary information on emerging unfavorable glycemic patterns and serve as a behavioral feedback tool within a structured follow-up. The proposed approach remains investigational as direct prospective evidence is lacking, validated CGM thresholds for defining metabolic relapse in obesity without diabetes are absent, and abnormal CGM findings should be treated as screening signals requiring confirmation rather than diagnostic endpoints. Future research should prioritize the prospective evaluation of CGM-guided post-discontinuation pathways, determine their incremental value beyond conventional monitoring, define clinically meaningful CGM metrics and thresholds for individuals with obesity without established diabetes and assess clinical effectiveness and cost-effectiveness before CGM-guided care can be recommended in routine obesity care.
